# Transient Interactions of α‑Synuclein N- and C‑Termini

**DOI:** 10.1021/acschemneuro.6c00108

**Published:** 2026-03-30

**Authors:** Lei Ortigosa-Pascual, Noemi Ferrante Carrante, Katja Bernfur, Katarzyna Makasewicz, Emma Sparr, Sara Linse

**Affiliations:** † Department of Biochemistry and Structural Biology, Lund University, Lund 221 00, Sweden; ‡ Department of Chemistry, King’s College London, London SE1 1UL, U.K.; § Division of Physical Chemistry, Lund University, Lund 221 00, Sweden; ∥ Department of Chemistry and Applied Biosciences, Institute for Chemical and Bioengineering, ETH Zurich, Zurich 8093, Switzerland

**Keywords:** self-assembly, coassembly, intermediates, peripheral membrane protein, cooperativity

## Abstract

α-Synuclein (αSyn) is a neuronal protein predominantly found at the synapse, involved in vesicle trafficking. αSyn aggregates are also the main component of Lewy bodies, the hallmarks of Parkinson’s disease. Interactions between the N- and C-termini of αSyn play crucial roles in its behavior in solution, membrane binding, and aggregation. Studying these interactions provides valuable insights into the physiological and pathological functions of αSyn. Here, we employed photoinduced cross-linking of unmodified proteins (PICUP) to identify the transient contacts of αSyn in different conformational states. By using tyrosine-to-phenylalanine mutations to block the reactivity of specific amino acids, we establish key cross-links in each state. In solution, we identify internal contacts between the N- and C-termini of monomers, as well as intermonomer contacts between C-termini in oligomers. When αSyn is bound to membranes, the internal cross-linking is blocked, while the cross-linking between C-terminal regions persists. In fibrils, cross-linking is significantly reduced, primarily occurring between the C-termini of adjacent monomers. This work highlights the effectiveness of PICUP for reporting on the transient contacts involved in αSyn self-assembly and its coassembly with lipid membranes, while providing a streamlined protocol that opens avenues for studying protein–protein interactions for a wide range of systems.

## Graphical abstract

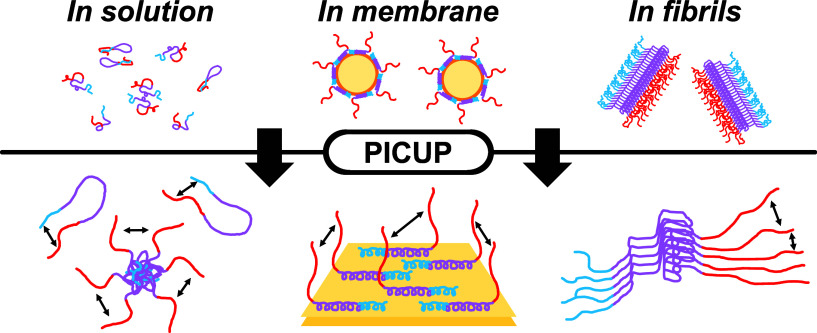

## 1 Introduction

α-Synuclein (αSyn) is a neuronal protein associated with synaptic vesicle trafficking. Its aggregation has been linked to several neurodegenerative diseases, most notably dementia with Lewy bodies, multiple system atrophy, and Parkinson’s disease. For the latter, αSyn has been detected in Lewy bodies and Lewy neurites in the brains of Parkinson’s disease patients^1^ and even single nucleotide substitutions in the αSyn-coding gene have been linked to sporadic and familial Parkinson’s disease.^2^ αSyn has since been the target of many studies and has been thoroughly characterized in terms of membrane binding, aggregation and structure.^3–9^

αSyn is a 140-amino acid protein of the synuclein family with a highly asymmetric charge distribution (Figure 1A). The sequence can be divided into three regions based on αSyn’s fibril formation propensity, an N-terminal (Nt) tail, a hydrophobic fibril core, and a C-terminal (Ct) tail. The Nt has a net positive charge at physiological pH, with 10 lysine and 8 acidic residues. Its high conservation throughout the protein’s evolution suggests that the Nt plays a critical role in the physiological functions of αSyn.^10^ Additionally, the Nt being a hotspot for genetic mutations associated with familial Parkinson’s disease variants underscores its pathological significance.^2,11–16^ The central region, or fibril core, has a high content of hydrophobic residues, which play a key role in the stacking of monomers into β sheets to form amyloid fibrils. Residues 1–95 include seven imperfect repeats of a motif with the consensus sequence KTKEGV. These repeats have been shown to contribute to the lipid membrane binding of the protein both in vivo^17^ and in vitro.^18^ The Ct, with 15 acidic amino acids, is strongly negatively charged at neutral pH and its p*K*_a_ values are upshifted upon fibril formation,^19^ which reduces the electrostatic repulsion in the fibril. The interaction between the oppositely charged N- and C-termini, be it within or between proteins, has been shown to play a key role in αSyn self-assembly.^20–24^

**Figure 1:**
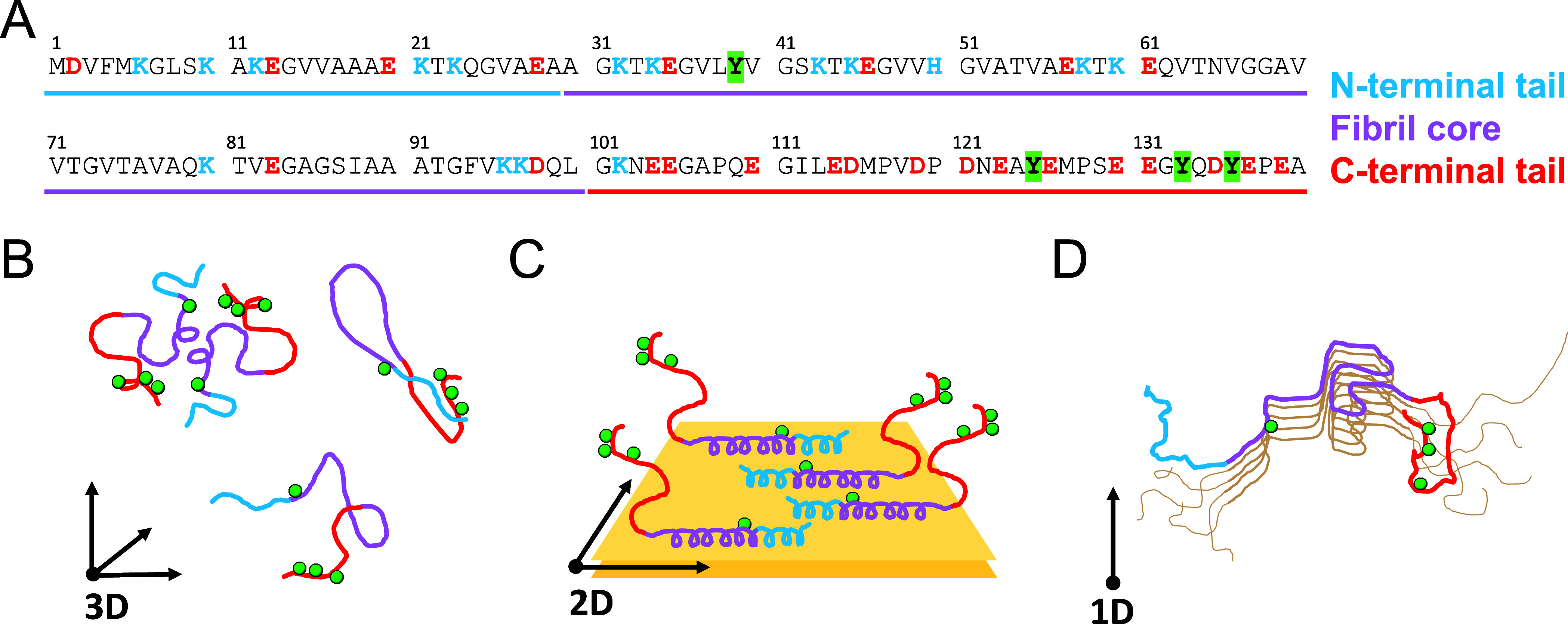
(A) αSyn sequence color coded to depict N-terminal tail (blue), fibril core (purple), and C-terminal tail (red). Negatively charged residues are shown in red, positively charged residues in blue, and tyrosines 39, 125, 133, and 136, main targets of the PICUP reaction, are highlighted in green. Cartoon representations of αSyn in solution (B), bound to membrane (C) and in fibril structure (D). The color coding represents the N-terminal tail (blue), the fibril core (purple) and the C-terminal tail (red), with the tyrosine residues, targets of the PICUP reactions, depicted as green spheres. The relative distribution of αSyn molecules can be described in 3D (B), 2D (C), and 1D (D) depending on the system depicted.

Being an intrinsically disordered protein, monomeric αSyn does not adopt a particular structure, but rather exchanges rapidly between different conformations^25^ (Figure 1B). This has been found to be the case also in vivo.^26^ When αSyn binds to membranes, the first ca. 100 residues adopt an amphipathic α-helix structure, with the remaining amino acids maintaining a disordered conformation^27^ (Figure 1C). The helix sits on the headgroup layer of the lipid membrane, oriented parallel to the membrane plane, causing only small perturbations of the lipid hydrocarbon packing.^3^ Furthermore, it was demonstrated that the binding takes place when the membrane contains anionic lipids, while there is no detectable binding to membranes containing only zwitterionic lipids.^27^ The disordered C-termini extend toward the solution forming a polymer brush of an estimated thickness of ca. 6 nm.^3^ Various αSyn binding modes have been observed, involving a different number of residues in the α-helix formation depending on the available lipid surface.^28,29^ Recent works have also shown that αSyn binding to membranes occurs with positive cooperativity, meaning that the affinity of a protein is higher for a membrane with a previously bound αSyn molecule over a bare membrane,^30^ and this property is robust over several solution conditions.^31^ The importance of αSyn–lipid interactions is also inferred for the disease-associated state, as Lewy bodies show a high content of both.^32^ Therefore, improving our understanding of the αSyn–lipid interactions is crucial to shed light on the αSyn activity in membrane remodeling and vesicle trafficking as well as its role in pathogenesis.

αSyn is an amyloid protein with a propensity to aggregate into fibrils. Although different morphs of αSyn fibrils exist,^23,33,34^ they all share the stacking of monomers forming extended β-sheets parallel to the fibril axis^33,35^ (Figure 1D). This aggregation is driven by hydrophobic interactions in the fibril core, while the negatively charged Ct remains disordered and extends into solution, forming a “fuzzy coat” with elevated p*K*_a_ values, meaning there is significant charge regulation upon fibril formation.^19^ The aggregation process follows a nucleation-dependent mechanism, which starts with the assembly of two or more monomers.^36^ These species, referred to as oligomers, are transient, and have a higher tendency to dissociate back into monomers than to convert and nucleate into fibrillar structure.^37,38^ After nucleation, the species formed are more prone to grow than to dissociate, and subsequent secondary nucleation leads to the autocatalytic nature of the aggregation process. Interestingly, αSyn oligomers have been shown to be toxic,^39,40^ as is the case for other amyloid proteins.^41–44^ However, their heterogeneity, transient nature, and low concentration relative to monomers and fibrils make oligomers challenging to study.^37,45–47^

One of the many methods commonly used for oligomer studies is cross-linking.^48^ If the cross-linking reaction is rapid enough, one can capture the transient species by forming covalent bonds in noncovalent complexes. Photoinduced cross-linking of unmodified proteins (PICUP)^49^ has been used to study amyloid oligomers of insulin,^50^ tau^51^ or amyloid β,^52^ among others. PICUP is a redox reaction that is initiated by exposing the metal complex ruthenium­(II) tris-bipyridyl cation (Ru­(bpy)) to light. The excited state of Ru­(bpy) donates an electron to ammonium persulfate (APS), which turns into its oxidant form. This attracts an electron from a nearby amino acid to form a reactive radical, which subsequently reacts with another residue to form a single covalent bond.^49^ Being a photoinduced cross-linking method, PICUP is easier to control compared to other chemical cross-linking methods, and can be achieved in considerably shorter times, having been used efficiently with reaction times even below one second.^49,52–56^ As a redox reaction, it has the potential to react with different amino acids, but it has shown to have a strong preference for tyrosine and tryptophan side chains.^50,52,54,57^ αSyn contains four tyrosine residues at positions 39 (Y39), 125 (Y125), 133 (Y133), and 136 (Y136) (Figure 1), making it a suitable protein to study with PICUP.^56,57^ Finally, the fact that it forms a single covalent bond means that the amino acids must be within covalent bonding distance during the lifetime of the radical to be cross-linked. This makes PICUP a highly valuable tool for gaining information about the structure of cross-linked species.

Recently, we developed a reaction chamber to optimize the method and observe cross-linking at reaction times as short as 1 ms.^56^ PICUP of αSyn in solution depends highly on the lighting time used to trigger the reaction.^56^ Using different experimental conditions, we demonstrated the PICUP product of αSyn in solution to be the consequence of oligomer formation and not of diffusion into close proximity. In the current study, we use this knowledge to study the transient interactions between αSyn monomers in different states. When αSyn is free in solution, it interacts with its neighboring proteins in three dimensions (Figure 1B). When bound to membranes, monomers are constrained to a two-dimensional plane (Figure 1C). Finally, when aggregated into fibrils, αSyn monomers are stacked on a one-dimensional axis (Figure 1D). By performing PICUP of αSyn in these different states, we aim to elucidate the effect of the dimensionality of the system on the cross-linking of the protein. The study is performed at mildly acidic pH (pH = 5.5), mimicking the environment found in lysosomes and endosomes, where secondary nucleation is most prominent.^36^ Using a combination of various tyrosine to phenylalanine mutants to inhibit the cross-linking capacity of different residues in αSyn, we identified the main participants of PICUP cross-linking in the different systems. This allowed us to identify two main groups of cross-links in solution: Y39–Ct, which happens intramolecularly, representative of the N–C-termini interaction in monomers in solution; and intermonomer Ct–Ct, which is the main source of PICUP-visible oligomers. When bound to membranes, the internal cross-linking is blocked, while the Ct–Ct cross-linking between monomers remains, presumably due to the high packing density of C-termini sticking into solution. Finally, in fibrils, αSyn can be cross-linked only via Ct to neighboring monomers, reducing its cross-linking significantly. This study sheds light into the contacts between and within αSyn molecules that PICUP can capture and establishes the technique as a powerful method for probing transient protein–protein interactions.

## 2 Results

### 2.1 Cross-Linking in Solution

#### 2.1.1 WT αSyn in Solution

PICUP of αSyn in solution leads to the formation of species larger than monomers.^56^ This can even be observed in samples where monomeric αSyn has been isolated, due to the fast formation of PICUP-reactive oligomers.^56^ The outcome of cross-linking depends on the reaction time, which is controlled by the light exposure of the sample. This way, when the sample is exposed to light for a short duration (i.e., 10 ms), only one new band appears in the SDS-PAGE gel, corresponding to the migration of a dimer (Figure 2A, top, lane “10 ms”). Increasing the reaction time increases the chances of cross-linking bigger species. However, it also has the risk of cross-linking species more than once, either between monomers or within the monomer, leading to products with more complex morphologies. This causes the formation of more than one band per oligomer order. When many bands with slight variation in electrophoretic mobility are formed, the individual bands merge together to form a diffuse long band, as seen when performing the reaction for 1s or more (Figure 2A, top, lane “100 s”).

**Figure 2:**
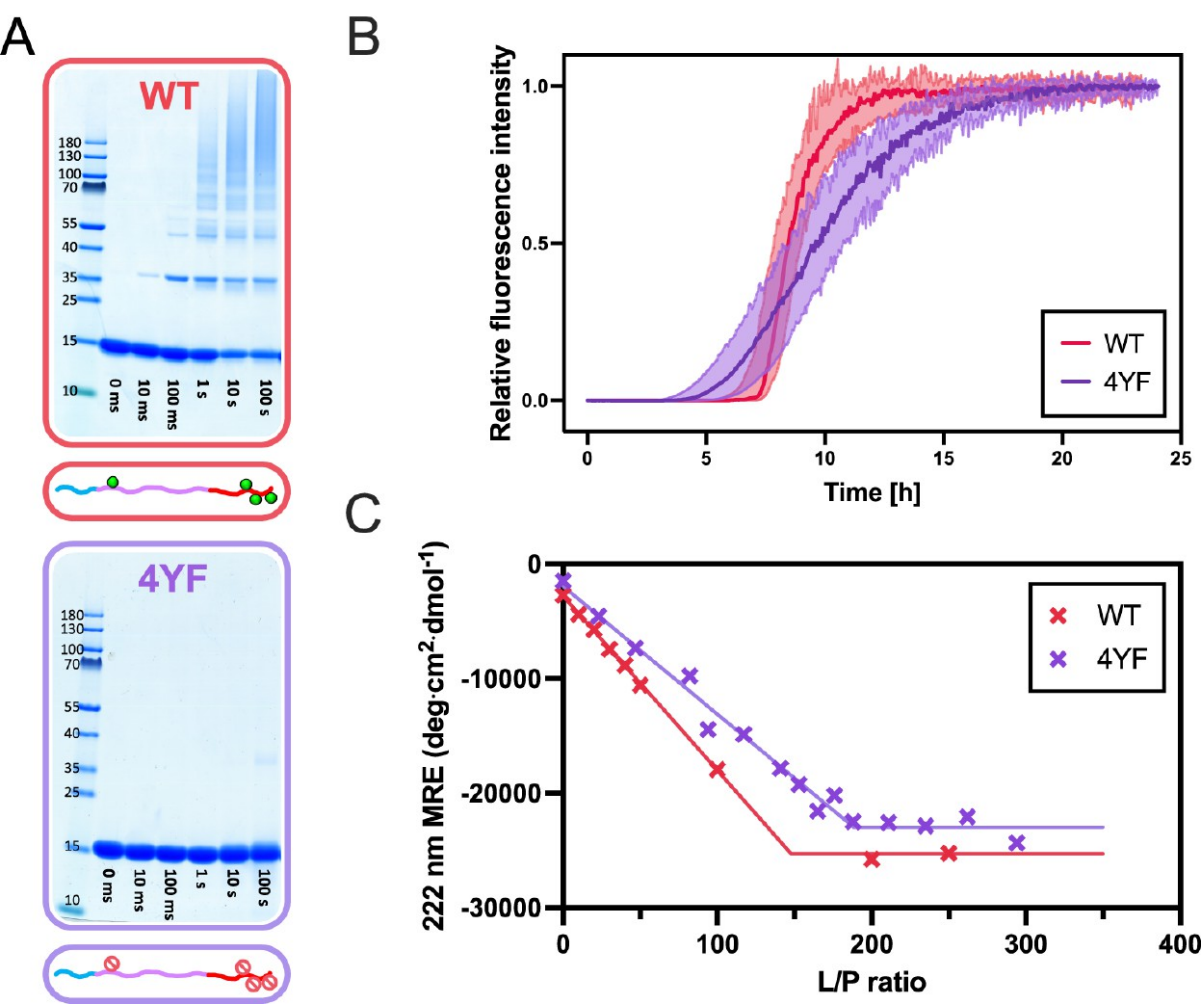
Role of tyrosine residues in PICUP, aggregation and lipid binding of αSyn. (A) PICUP of WT (top) and 4YF (bottom) αSyn in solution. The reaction was performed for 0 ms, 10 ms, 100 ms, 1 s, 10 s, and 100 s. Full gels can be seen in Figure S1. (B) Aggregation kinetics of WT (red) and 4YF (purple) αSyn monitored by ThT fluorescence. (C) Lipid binding of WT (red) and 4YF (purple) αSyn measured by CD. Mean residue ellipticity (MRE) at 222 nm was measured as a function of L/P for both proteins (cross marks). The line shows the trend of the MRE as a function of L/P, with the intersection indicating the L/P value at which no more α-helix is formed (WT ≈ 150, 4YF ≈ 190).

#### 2.1.2 Tyr → Phe Mutation

Previous studies have identified tyrosine (Tyr, Y) as the main source of cross-linking of αSyn via PICUP.^52–54,57^ Phenylalanine (Phe, F), retaining the aromaticity but lacking the hydroxyl group of Tyr, seems to have a considerably lower efficiency of cross-linking.^53,58^ With the aim to eliminate the cross-linking capacity of αSyn, we therefore expressed a mutant form of the protein with the four Tyr residues (Y39, Y125, Y133, and Y136) mutated to Phe, named 4YF.

When performing PICUP with the 4YF mutant under the same conditions as those for the wild type (WT) αSyn, we indeed observed close to zero cross-linking (Figure 2A, bottom). A very faint band can be seen when the reaction is performed for as much as 100 s, which can thus be attributed to the very low efficiency of nontyrosine cross-linking. In order to evaluate whether the lack of cross-linking of 4YF is due to a drastic change in protein behavior, the mutant protein was subjected to aggregation and vesicle binding studies (Figure 2B,C). The thioflavin T (ThT) fluorescence intensity as a function of time shows that 4YF aggregates on a similar time scale to WT αSyn (Figure 2B), albeit the shallower transition of the mutant may reflect a smaller dominance of secondary nucleation.^59^ The conformational change from disordered to α-helical structure αSyn undergoes upon binding to vesicles can be probed by means of CD spectroscopy,^27^ providing an estimation of the lipid-to-protein ratio (L/P) above which no more protein binds to the vesicles, here referred to as saturation point. Figure 2C shows that the binding behavior of the 4YF mutant to the vesicles is similar to that of WT αSyn, with saturation points of L/P ≈ 150 and L/P ≈ 190 for the WT and 4YF proteins, respectively. Taken together, these results indicate that the observed differences in ability of the 4YF mutant to cross-link are due to the lower reactivity of phenylalanine rather than changes in the overall self- and coassembly properties of the protein caused by the mutations.

#### 2.1.3 Role of Each Tyrosine in αSyn PICUP

To determine the role of each Tyr residue in the cross-linking of αSyn in solution, four different single-amino acid Tyr → Phe mutants were purified and used in PICUP studies in the same manner as the wild-type: Y39F, Y125F, Y133F, and Y136F. The three C-terminal mutants (Y125F, Y133F, and Y136F) showed the same behavior as WT αSyn, with the formation of a diffuse long band under longer reaction times (Figure 3, top row). However, Y39F shows a strikingly different pattern (Figure 3, bottom left). Increasing the reaction time of Y39F leads to the formation of new bands of slower migration, but not to the point where the bands blend into a diffuse long band. Rather, the results reveal a single band for each oligomer order. This points at Tyr39 generating additional cross-links for the WT αSyn, leading to a more diverse range of cross-linked products and, thus, more gel bands.

**Figure 3:**
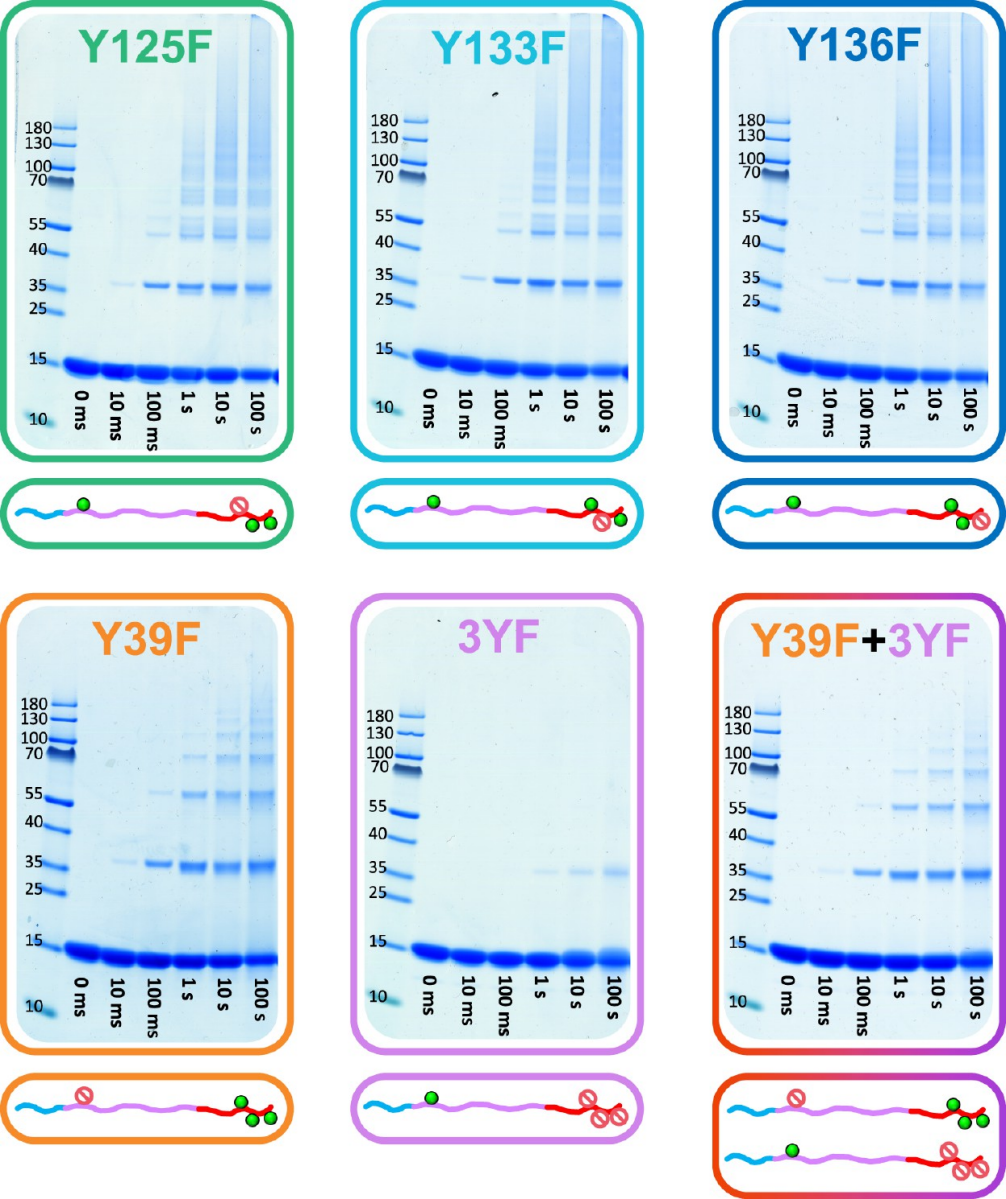
PICUP of αSyn with various Tyr → Phe mutations. PICUP was performed for αSyn mutants Y125F, Y133F, and Y136F (top row) as well as Y39F (bottom left) to evaluate the effect of single amino-acid Tyr → Phe mutations in PICUP. In order to elucidate the role of Tyr39, a mutant with the three C-terminal Tyr (Y125, Y133, and Y136) mutated to Phe was generated, named 3YF (bottom middle). Finally, a 50:50 mixture of αSyn mutants 3YF and Y39F was also tested (bottom right). The reactions were performed with lighting times of 0 ms, 10 ms, 100 ms, 1 s, 10 s, and 100 s. Full gels can be seen in Figure S4.

To further evaluate the role of Y39, a fifth mutant was expressed, with Y125, Y133, and Y136 all mutated to Phe. This mutant, named 3YF, contains Y39 as the only potential source of cross-linking. The cross-linking of this mutant led to the formation of dimer bands only and only after a very long reaction time (100 s) (Figure 3, bottom middle).

#### 2.1.4 How Does Y39 Contribute to Heterogeneity of PICUP Products?

Y39 could be leading to a higher diversity of cross-linked morphologies either by participating in monomer–monomer cross-links or by leading to internal cross-linking within a monomer. To distinguish between these two possible contributions, we prepared a reaction with a 50:50 mixture of Y39F and 3YF. As these mutants contain either the C-terminal tyrosines or Y39, respectively, the ability of the monomer to intramolecularly cross-link is abolished, while allowing for monomer–monomer cross-linking between the Y39 and the C-terminal tyrosines. Strikingly, cross-linking of this sample led to the formation of bands akin to those observed for Y39F alone (Figure 3, bottom right).

### 2.2 Cross-Linking of Protein Bound to Lipid Vesicles

While proteins in the bulk interact with each other in 3D, the binding of αSyn to a lipid membrane surface places the proteins in a 2D plane, which alters their potential interactions. When αSyn is cross-linked in the presence of vesicles, the outcome changes drastically depending on the relative concentration of protein and lipids in terms of the L/P (Figure 4A). Increasing the L/P leads to the formation of more defined bands. This may be due to both a transition to a system with fewer degrees of freedom (2D-like), or a change in structure of the protein when binding to the lipid membrane. The change to more defined bands from solution to lipid bound becomes most pronounced at L/P ≥ 150, which happens to be close to the saturation point for WT αSyn (Figure 2C). This strongly indicates that the bands observed at both L/P = 200 and 250 represent the cross-linking of fully bound αSyn, and that the transition we observe between L/P = 0 and 150 can be related to a change in the proportion of protein that is free in solution and bound to lipid membranes.

**Figure 4:**
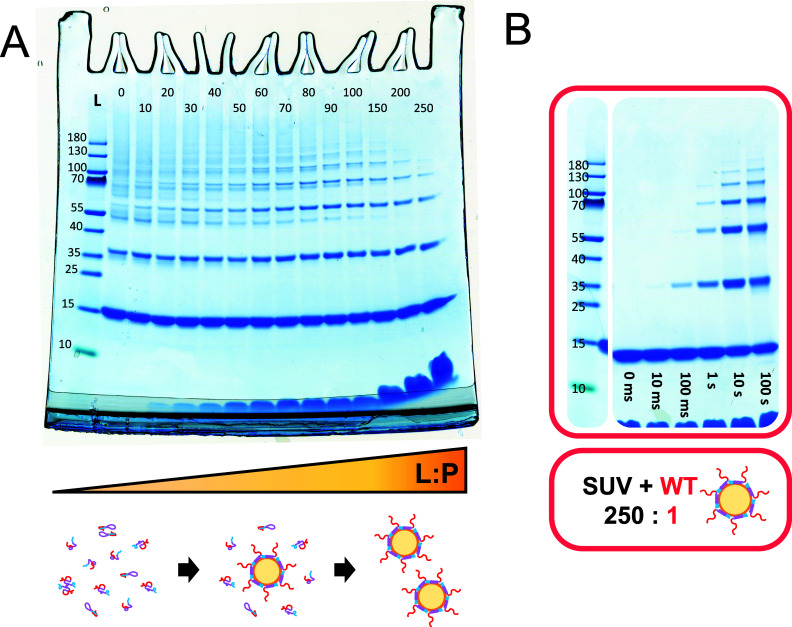
Effect of lipid binding on PICUP of αSyn. (A) PICUP was performed to αSyn for 1 s in the presence of SUVs at different L/P (0–250). (B) At L/P = 250, where all the αSyn is bound to the vesicles, PICUP was performed for 0 ms, 10 ms, 100 ms, 1 s, 10 s, and 100 s. Full gel of figure B can be seen in Figure S1. The lipids are observed at the bottom of the gel, stained by Coomassie.^101^

To study the protein bound to vesicles, we used a system with a L/P of 250, which is above the saturation points of both WT and 4YF (Figure 2C), meaning that no further binding occurs even if more vesicles are added. Performing PICUP at different lighting times for WT at L/P = 250 shows that increasing the lighting time does not generate new bands other than the well-defined ones (Figure 4B). Strikingly, these well-defined bands have the same molecular weight as those observed for the Y39F mutant in solution (Figure 3A). This is more clearly seen in the full gels, available in Supporting Information (Figures S1 and S2).

The same experiments at saturating L/P (L/P = 250) were run for the different mutants (Figure S2). All of the single point mutants, regardless of their cross-linking in solution, displayed similar behavior as that of WT αSyn when bound to vesicles. Interestingly, vesicle-bound Y125F shows less bands than WT αSyn under the same conditions (Figure S2). Additionally, Y125F seems to require a higher L/P than the WT for the extra bands to disappear (Figure S3). Mutants 4YF and 3YF were also evaluated under the same conditions (Figures S1 and S4). 4YF showed absolutely no bands when bound to vesicles, and 3YF behaved the same as in solution, forming mostly dimers.

### 2.3 Cross-Linking in Fibrils

When αSyn aggregates into fibrils, the monomers stack together, forming β-sheets parallel to the fibril axis. This way, the αSyn monomers are stacked along a 1D axis, reducing the available interaction partners of each monomer compared to the in-solution and lipid-bound systems. As previously reported, performing PICUP on fibrillated αSyn leads to the formation of almost no bands.^56^ In fact, when PICUP is performed at different times during the aggregation process, the cross-linked species can be seen decreasing in concentration (Figure 5A). This observation applies to all the Tyr → Phe mutants, too (Figure 5B). All single point mutants (Y39F, Y125F, Y133F, and Y136F) show a behavior that is undistinguishable from that of WT αSyn. As previously reported, two bands below the cross-linked dimer band can be observed upon aggregation of WT αSyn, which are independent of cross-linking.^56^ This same phenomenon is observed for all mutant αSyn variants (Figure 5B).

**Figure 5:**
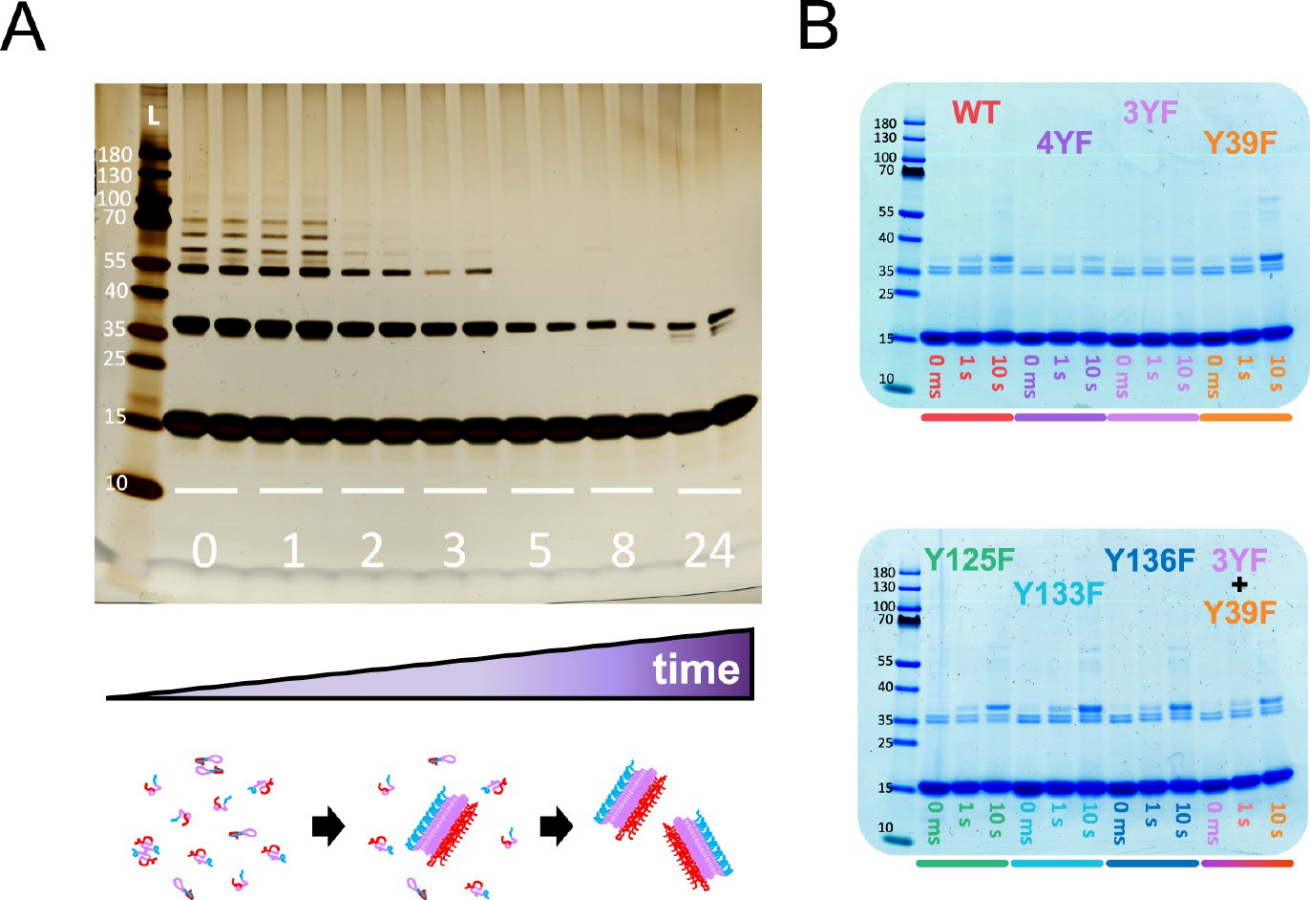
PICUP of αSyn on fibrils. (A) PICUP at 1 s lighting time of WT αSyn over aggregation time. The numbers indicate the time of aggregation in hours. Sample was collected before the start of aggregation (“0”), middle of lag phase (“1”), start of exponential phase (“2”), middle of exponential phase (“3”), end of exponential phase (“5”), final plateau (“8”), and late after full aggregation (“24”). Reproduced from.^56^ Copyright 2023 American Chemical Society. (B) PICUP of fully fibrillated WT αSyn as well as all the Tyr → Phe mutants of αSyn used in this study. Sample was cross-linked for 0 ms, 1, and 100 s.

When all Tyr are removed (mutant 4YF), the cross-linked dimer band becomes weaker, but it does not completely disappear, suggesting a non-Tyr amino acid is cross-linking in fibril form too. In fact, when comparing 4YF and WT αSyn, the reduction of the cross-linking product caused by the removal of all Tyr is much lower in fibrils than it is in solution. The fact that 4YF and 3YF show a similar decrease in cross-linked dimer band, moreover, points at the possibility of Y39 not taking part in cross-linking in fibrils.

## 3 Discussion

Studies of the self- and coassembly of αSyn are crucial in order to fully understand its native and pathological functions. Here, we show the potential and versatility of PICUP as a tool to study transient interactions between αSyn molecules in different environments. We find that the dimensionality of the system studied alters the cross-linking pattern, and parallel studies in different geometries provide more information about the positioning of αSyn (Figure 7).

### 3.1 Cross-Linking in Solution

The presence of clear bands of defined size, in combination with diffuse long bands, suggests the coexistence of at least two groups of cross-linked species and reflects on the complexity of the cross-linking that occurs upon long reaction-time.^56^ By mutating Tyr to Phe, we aimed to modify the reactivity of each residue, allowing us to assess the contribution of each position to the cross-linking pattern.

#### 3.1.1 Tyr → Phe Mutations Decrease αSyn PICUP Reactivity

Substituting all Tyr residues with Phe (mutant 4YF), while keeping aggregation and lipid binding properties similar, severely decreased the level of cross-linking of αSyn. The presence of a faint band at a 100 s lighting time suggests that a residue other than tyrosine may cross-link with very low reactivity. This could be a residue present in WT αSyn, or the newly added Phe residues, which do show weak cross-linking reactivity.^53^

When the three Tyr residues of the Ct were replaced (3YF), leaving only Y39 available to cross-link, only dimer bands were observed. These bands show higher intensity than those observed for the 4YF mutant, suggesting that Y39 is involved in the formation of these cross-linked species. The fact that 3YF only forms dimers implies that a Tyr cannot cross-link more than one other Tyr, although dityrosine has been reported to react with a third Tyr.^60^ However, the tight distance restriction of PICUP makes a triple-Tyr cross-link highly unlikely.

#### 3.1.2 Y39 Is Responsible for Diffuse Bands in PICUP of αSyn

When the four Tyr residues of αSyn were mutated to Phe one at a time, clear differences were observed (Figure 3). While mutating either of the Ct residues did not lead to any detectable changes, the mutation of Y39 led to the disappearance of the diffuse bands with lower mobility. This indicates that Y39 is responsible for increasing the complexity of the cross-linking pattern, and since a molecule of a defined size can migrate faster or slower depending on its shape, this leads to the formation of new bands. Figure 6 illustrates the different ways that the addition of Y39 could increase the complexity of the species resolved by SDS-PAGE.

**Figure 6:**
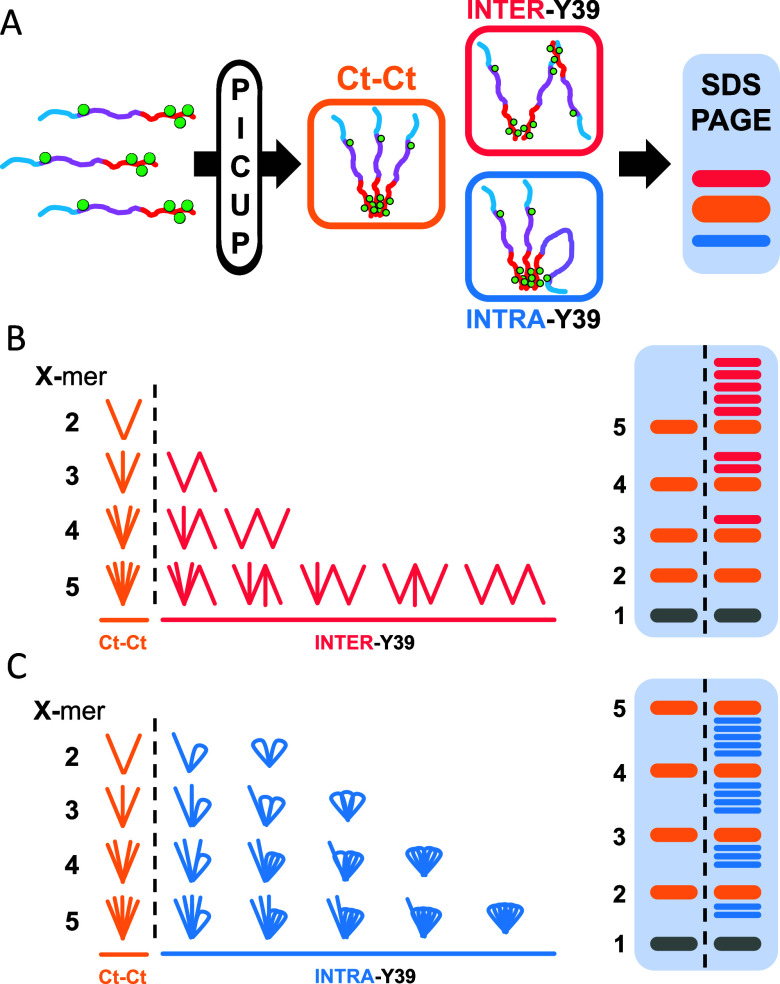
Schematic representation of the possible effects of Y39 in cross-linking of αSyn. (A) If cross-linking of αSyn occurred only via the Ct Tyr residues, we would form what we here refer to as “bouquet” shape (“Ct–Ct”, orange). If Y39 participated on intermolecular cross-linking, it would lead to the formation of more branched morphologies (“INTER-Y39”, red). If Y39 instead participated in intramolecular cross-linking, it would cause the “folding” of a monomer onto itself (“INTRA-Y39”, blue). If one were to run these three morphologies in an SDS-PAGE gel with sufficient resolution (A, right), they would be separated due to their differences in conformation, where the more compact shape would run faster (“INTRA-Y39”) and the more expanded one would run slower (“INTER-Y39”). While Ct–Ct cross-linking would lead to a single shape per oligomer order, both intermolecular (B) and intramolecular (C) Y39 cross-linking would give rise to more possible morphologies the higher the oligomer order. These would be seen in the SDS-PAGE as bands above or below the one formed by the Ct–Ct bouquet shape, respectively.

Given the proximity of residues Y125, Y133, and Y136, one would expect proteins cross-linked via any of them to be similar enough in shape to be indistinguishable by SDS-PAGE. This is supported by the similarity between their Tyr → Phe mutants (Figure 3). We thus refer to the three of them together as “cross-linking the Ct”, leaving three possible cross-links between monomers: Y39–Y39, Y39–Ct, and Ct–Ct.

In a system where only Ct–Ct cross-links occur, monomers would be cross-linked together in what we call a “bouquet” shape (labeled “Ct–Ct”, orange) (Figure 6A). In this system, each oligomer order would give rise to a single shape and, thus, a single band in SDS-PAGE (i.e., one for dimer, one for trimer, etc.). The addition of Y39 to the system could increase the complexity of αSyn cross-linking in three ways. First, if Y39 participated in intermolecular cross-linking (“INTER-Y39”, blue), this would lead to the formation of cross-linked oligomers that would be more extended than the “bouquet” ones. These more extended species would migrate less in SDS-PAGE gel and would form a band above the “bouquet” band (Figure 6A). Second, if Y39 participated in intramolecular cross-linking (“INTRA-Y39”, red), this would cause the monomers to fold over themselves, creating more compact oligomers. These would migrate further than the Ct-Ct oligomers, forming a band below the “bouquet” band (Figure 6A, right). Third, Y39 could be contributing to the complexity of the cross-linking pattern by participating in both inter- and intramolecular cross-linking.

The effect of inter- and intramolecular cross-linking of Y39 for different oligomer orders is represented in Figure 6B,C, respectively. While the system with only Ct–Ct cross-linking would lead to a 1 oligomer = 1 band pattern, if Y39 is involved in either inter- or intramolecular cross-linking, it would lead to ≥1 band per oligomer. In either case, the higher the oligomer order, the more possible morphologies of the cross-linked species, explaining why the diffuse behavior observed at high lighting times is more prominent at higher molecular weights.

#### 3.1.3 Y39 Participates in Intramolecular Y39–Ct Binding Only

By mixing mutants Y39F and 3YF, we create a system where Y39-mediated intermolecular cross-linking is allowed, thus having the possibility of forming Y39–Y39, Ct–Ct, and interY39–Ct cross-links. However, intraY39–Ct cross-linking cannot occur. Strikingly, PICUP of the Y39F and 3YF mixture looks very similar to that of Y39F alone (Figure 3, bottom right). This means that removing the ability of Y39 to do intramolecular cross-linking has the same effect as removing the ability of Y39 to cross-link at all. This finding shows that the only role of Y39 in the cross-linking of WT αSyn is intramolecular Y39–Ct contact (intraY39–Ct). Additionally, this suggests that during PICUP of WT αSyn no observable intermolecular Y39–Ct nor Y39–Y39 cross-links form, as those would have led to the formation of more bands than observed for Y39 alone. While intermolecular Nt–Ct interactions could be expected given their opposite charges, previous studies by NMR spectroscopy have reported that intramolecular Nt–Ct interactions dominate the system at αSyn concentrations below 250 μM,^61^ supporting our results. In short, the only contribution of Y39 to the cross-linking of αSyn is that depicted in Figure 6C.

Given the observation of cross-linked dimers with the 3YF mutant, attributed to Y39–Y39 cross-linking above, it seems that Y39–Y39 can form in the absence of other cross-linkable residues, but not when the three Ct Tyr are present. The fact that 3YF needs more light to form dimers than WT or mutants containing the Ct Tyr do suggests Y39–Y39 to be a less favored cross-link. The Y39–Y39 cross-link might not form in WT αSyn for several reasons. First, intraY39–Ct may have a strong preference over Y39–Y39. In addition, Y39–Y39 cross-links in WT αSyn may occur only between monomers that already contain a Ct–Ct cross-link, which would not alter the migration of the oligomer.

The obtained results imply that two cross-links are preferentially formed for αSyn in solution: intraY39–Ct and Ct–Ct (Figure 7, left). These cross-links are most likely favored by transient interactions between monomers.

**Figure 7:**
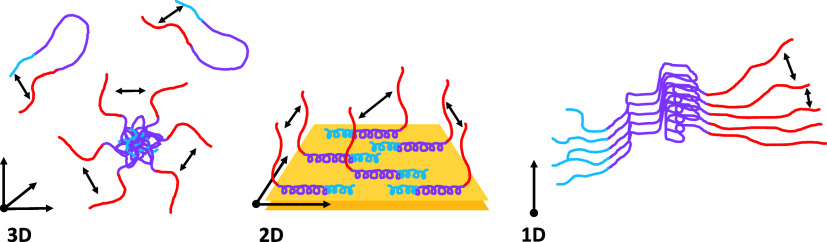
Cartoon of transient interactions of αSyn identified with PICUP, and their dependence on the dimensionality of the system. In solution (3D), PICUP reports on internal Ct–Nt interactions, as well as Ct interactions of oligomeric species. When bound to lipids (2D), only Ct–Ct interactions are detected between proteins. When αSyn is fibrillated, only the Ct–Ct cross-linking is detected, albeit with less frequency than in the other two systems.

#### 3.1.4 IntraY39–Ct Cross-Linking: αSyn’s Self-Chaperoning Ct

The finding that intraY39–Ct is one of the main cross-links obtained with PICUP of αSyn comes as no surprise given that it is statistically favored over intermolecular cross-links due to the close proximity of Nt and Ct within an αSyn molecule compared to the average distance between separate αSyn monomers. On top of that, intraY39–Ct cross-links reflect an interaction between the N- and C-termini of αSyn. Previous studies have shown that monomeric αSyn adopts a more compact conformation than that of a random coil, due to long-range interactions between its Nt and Ct.^62,63^ Internal Nt–Ct attraction has been discussed to disfavor the interactions between NAC regions of different monomers, preventing protein aggregation.^21,24,62–66^ Binding of Ca^2+^, Cu^2+^, or polyamines to the Ct may lead to higher exposure of the Nt and NAC domains, accelerating the aggregation.^67–69^ Truncations of Ct occur in vivo both in healthy and diseased brains,^70–73^ leading to a more extended αSyn conformation,^63^ faster fibril formation,^24,74–79^ higher toxicity^80–82^ and increased mitochondrial damage.^63^ In addition, Ct truncation increases the interaction of αSyn with lipid membranes and molecular chaperones by freeing the Nt involved in those interactions.^63,83^ In short, the Ct plays a self-chaperoning role in the self- and coaggregation of αSyn by interacting with the Nt.

The finding that intraY39–Ct is one of the two main cross-links obtained with PICUP of αSyn would also be expected based on the previously found interactions between Nt of monomers and the exposed Ct of fibrils. The screening of the monomer–fibril interaction by salt as seen in experiment and simulations imply that electrostatic interactions are a main driving force.^21^ This is supported by the observation that the addition of salt, while not affecting the pattern of cross-linked bands, decreases the intensity of the bands formed (Figure S5). However, it has been discussed that not only charges, but interactions between aromatic residues play a key role in the Nt–Ct interaction.^84^ Internal Nt–Ct interactions as observed by NMR and in simulations^21,22^ imply that Y39 and Ct will be in close enough proximity for PICUP to cross-link. Furthermore, dityrosine Nt–Ct cross-linked monomers obtained via oxidation, as well as PICUP cross-linked oligomers, have been shown to inhibit αSyn aggregation,^57,85^ which can now be explained with intraY39–Ct cross-linking. The finding of internal Y39–Ct cross-linking reflects on the key role of Ct in monomeric αSyn and shows PICUP to be a very useful tool to monitor this phenomenon.

#### 3.1.5 Ct–Ct Cross-Linking: Oligomeric αSyn’s Interactions

The Ct–Ct cross-linking must reflect the formation of aggregated species, e.g., oligomers. Given the proximity between the Nt and Ct in transient conformations of a monomer, the Y39–Ct contact may occur earlier than the Ct–Ct one. However, mutants without the ability to form internal Nt–Ct cross-linking also show oligomeric bands (Figure 3), suggesting that Ct–Ct cross-links do not depend on intraY39–Ct cross-linking. Thus, the oligomers captured via Ct–Ct are not artifacts due to a prior internal cross-linking of monomers. This means that the proximity between the C-termini is a characteristic feature of PICUP-visible oligomers (PvO).

αSyn oligomers, though heterogeneous, are on average more stable than those formed by other amyloid proteins,^37^ likely due to their higher β sheet content,^37,86,87^ predominantly in an antiparallel arrangement.^88–90^ This antiparallel structure is characteristic of kinetically trapped oligomers (KTO), highly stable species produced via defined protocols.^89,90^ Their stability is thought to stem from a high free energy barrier preventing their transition to the parallel β-sheet typical of fibrils.^87,90^ Conversely, parallel β-sheet oligomers may more readily transition into fibrils.^90^ While the exact contribution of the Ct region to oligomerization is debated, it clearly plays a significant part.^24,62,82^ Structural data show KTOs have a structured core with an unstructured Ct tail.^89,91^ Furthermore, while some KTOs considered to be off pathway^92^ have the Nt immobilized in the oligomer core, those exhibiting greater toxicity have the Nt accessible, this feature being an important part of membrane binding, disruption, and cell toxicity.^91^

In a previous study, we have shown that αSyn PvO appear quickly after monomer isolation, and their concentration dependence follows that of the monomer.^56^ Previous results indicate that αSyn PvO are in fast equilibrium with monomers, have low kinetic stability and a concentration determined by monomer depletion.^37,56^ It thus stands to reason that they are species on the pathway to fibrillar structure. We here find that the Ct of monomers must be accessible to each other in PvO. These oligomers can be obtained independent of an intraY39–Ct, as they are seen for both WT and Y39F. The fact that the intraY39–Ct does not restrict oligomer formation could point toward an oligomer structure that has both N- and C-termini exposed. Given that Ct-Ct is the main cross-link observed for PvO, it is likelier that the monomers are in a parallel β-sheet, as opposed to the KTO identified in literature, composed mostly of antiparallel β-sheet.^89,90^ However, we only observe a few monomers together. Thus, the observed bands could come from either small oligomers, for which there is no structural information available, or a small fraction of monomers within a bigger oligomer. Finally, most of the observations described here for αSyn oligomers could also apply to other small aggregate intermediates, which can also show antiparallel β-sheet structure and higher flexibility, reversibility and Nt availability than mature fibrils.^93,94^

### 3.2 Cross-Linking of Protein Bound to Lipid Vesicles

αSyn shows fewer bands when bound to lipid membranes compared to those in solution, suggesting that some residues that cross-link in solution are not in close proximity when the protein is bound to membranes. Helical projections of the α-helix adopted by the Nt of αSyn show Y39 on the solvent-exposed side of the helix,^9,95^ and available for potential cross-linking. 3YF is cross-linked only as dimers when bound to vesicles, which indicates that the formation of trityrosine is also structurally restricted in this state.

Adding more membranes (increasing the L/P) initially leads to the reduction of the number of bands. IntraY39–Ct cross-linking disappears as the L/P increases and the internal cross-linking is reduced (Figure 4A). The shadow below the dimer band in the lipid-free sample becomes weaker upon the addition of membrane and finally disappears at L/P = 150, around the saturation point. Adding more membrane above the saturation point does not lead to any further changes in cross-linking pattern, reflecting the cooperativity of the binding; the protein does not spread over the extra available membrane area but rather stays as densely packed and as clustered as at the saturation point.^9^ Similarly, the trimer for the lipid-free sample has two distinguishable bands, one with faster migration at ∼55 kDa and one with slower migration at ∼60 kDa. As L/P increases, the ∼55 kDa band disappears as the other one becomes stronger, until only the ∼60 kDa band is visible at L/P = 150. The band appearing at ∼55 kDa must thus originate from intraY39–Ct cross-linking of the protein in solution.

IntraY39–Ct cross-linking is thus impossible for αSyn bound to membranes (Figure 7, middle). To evaluate whether this is due solely to steric hindrance, we estimated the dimensions of key features of the vesicle-bound αSyn system (Section S6 of the Supporting Information). These calculations show that the average distance between two proteins at the membrane, and thus between two C-termini, is ∼73 Å, while the radius of gyration of the 40 residues composing the Ct tail in random coil conformation is ∼18 Å. At the same time, the distance from L100, where the Ct tail starts, and Y39 is of ∼90 Å, while the Ct tail can stretch as far as 145 Å. These results imply that, while crowded, there is room for the Ct tail to coil up or bend toward the membrane surface and come into contact with its own Y39 (Figure S6). Thus, the reason why we do not observe Y39–Ct cross-linking is more likely due to electrostatic repulsion between neighboring C-termini favoring a more extended state compatible with Ct–Ct cross-linking through random segment diffusion. However, the severe distance restriction of PICUP does not exclude that the cross-linking is reflective of specific interactions between C-termini. Moreover, due to the fluidity of a lipidic bilayer, αSyn proteins are not anchored in space and can move at the membrane, which may facilitate the interactions between protein molecules.

Notably, Y125F shows a behavior different from that of Y133F and Y136F (Figures S2 and S3). First, we observe that the extra bands that originate from the proteins in solution can be seen at higher L/P than for WT (Figure S3). This observation could be related to either a lower affinity to the SUVs or a higher exchange rate relative to WT. Second, the slightly fewer bands seen for Y125F compared to Y133F and Y136F suggests that Y125 is the main source of Ct–Ct cross-links in membrane-bound αSyn, supported by previous reports of Y125 being the most PICUP-reactive Tyr residue in the Ct.^58^ Finally, since it has been shown that Nt availability is crucial for oligomer activity and toxicity, oligomers with only Ct–Ct cross-linking might be more representative of toxic species.^91^ This means that performing PICUP in the presence of vesicles could be a useful tool to generate toxic oligomers with WT αSyn, without the intraY39–Ct that occurs in solution.

### 3.3 Cross-Linking in Fibrils

For WT αSyn fibrils, we obtain dimer bands only. 4YF αSyn shows fewer bands than WT; however, this difference is smaller than that in solution. The fact that 3YF shows the same intensity as 4YF suggests that Y39–Y39 cross-linking does not happen for WT αSyn in fibrils. Structures of αSyn fibrils show Y39 next to the fibril core, with the Y39 side chains of neighboring monomers π–π stacked face-to-face.^34,96^ This may suggest restricted rotation of the aromatic rings, limiting the possibility of covalent-bond formation. IntraY39–Ct cross-linking is also unlikely due to the long distance between the termini of the same monomer in the fibril.^34^ Mutants containing at least one of the Ct Tyr residues show as much cross-linking as WT αSyn does, whereas 4YF and 3YF show less. This suggests that Ct–Ct cross-linking still occurs between monomers in a fibril (Figure 7). Due to the proteins being stacked in a 1D axis, each monomer has fewer neighboring monomers to cross-link with, leading to the formation of mainly dimers, and for some of the mutants a faint trimer band is observed. In a previously identified αSyn fibril structure,^97^ Lys97 of neighboring monomers, where the random coil C-termini are anchored, are separated by ∼5 Å. The finding of much less Ct–Ct cross-linking compared to membrane-bound αSyn suggests that the interactions between C-termini are different in the two cases. This could be either due to the higher mobility of proteins on the membrane or due to specific interactions between C-termini in this state. PICUP may facilitate the understanding of the organization of αSyn on lipid membranes and shed light on the cooperativity of its binding.^30^ Given its sensitivity to proximity between monomers and their orientation, PICUP could also prove a valuable method to study differences in structure between fibril morphs.

### 3.4 Conclusions

The cross-linking of αSyn varies, depending on the dimensionality of the system (Figure 7). In solution (3D), a combination of the long-range interactions between the C- and N-termini of the monomer, and the interaction between C-termini of monomers in an oligomer, gives rise to a complex cross-linking pattern. When αSyn is bound to lipid membranes, confined to a 2D plane, the internal cross-linking is blocked, and we observe transient interactions between C-termini due to either chain diffusion of this mobile part or specific interaction between Ct tails. In fibrils, the interaction network is reduced to 1D, in which monomers can only interact with their direct neighbors, giving rise to less cross-linking. These measurements reflect on αSyn’s behavior as a monomer, as well as the transient interactions that occur in its self- and coassembly. This study underlines the value of PICUP to study the behavior of αSyn by capturing its transient contacts and presents a robust and accessible protocol for investigating interactions between and within proteins. By applying the framework presented in this study to different solution conditions and post-translationally modified variants of αSyn, PICUP presents itself as a very strong tool to understand the effects of modulators on the transient interactions of αSyn N- and C-termini.

## 4 Methods

### 4.1 α-Synuclein Expression, Purification, and Preparation

WT αSyn, as well as its mutant variants, was expressed in *E. coli* using a Pet3a plasmid with *E. coli*-optimized codons (purchased from Genscript, Piscataway, New Jersey). The protein was purified using heat treatment, ion-exchange, and gel filtration chromatography, as previously described by Ortigosa-Pascual et al.^19,55^ For the WT, 4YF, Y125F, Y133F, and Y136F proteins, no other bands were observed on the SDS-PAGE after purification (Figures S1 and S4). However, we note that mutants Y39F and 3YF showed a slight level of degradation in the form of a faint band below the monomer band, accounting for 1.8 and 1.7% of the sample, respectively (Figure S4). Each purified protein was aliquoted, freeze-dried and stored at −20 °C.

All experiments started with dissolving the aliquot of the protein variant of choice in 6 M GuHCl, followed by gel filtration on a 10 × 300 mm Superdex75 column (GE Healthcare). This step serves to ensure a fully monomeric sample and to exchange the buffer to 10 mM MES, pH 5.5, the initial conditions used for all our experiments.

The concentration of WT αSyn was determined by the absorbance at 280 nm using an extinction coefficient of ϵ = 5960 M^–1^ cm^–1^. Given that the different Tyrosine content of the mutants affects their 280 nm absorbance, but both the number of primary amines and peptide bonds in the protein remains the same, the concentration of all mutants was determined using *o*-phthalaldehyde (OPA) fluorescence intensity and 214 nm absorbance, using WT αSyn for the standard curve in both cases.^98,99^

### 4.2 Vesicle Preparation

Lyophilized lipids 1,2-dioleoyl-*sn-glycero*-3-phospho-l-serine sodium salt (DOPS) and 1,2-dioleoyl-*sn-glycero*-3-phosphocholine (DOPC) were purchased from Avanti Polar Lipids (Alabaster AL) and stored at −20 °C.

Small unilamellar vesicles (SUVs) were formed by extrusion as follows. The required amount of DOPC and DOPS was weighted and mixed in a glass vial in a molar ratio of 7:3 and dissolved in chloroform:methanol (9:1). The organic solvent was then evaporated by applying a gentle flow of nitrogen, and the vial was kept in a vacuum chamber overnight to remove any excess solvent from the lipid film. Lipids were dispersed in buffer containing 10 mM MES at pH 5.5 and vortexed for a few minutes to get a final lipid concentration of 8 mM. The lipid dispersion was extruded 23 times using an Avanti Mini Extruder (Avanti Polar Lipids) and 100 nm pore size filters. The filters were previously saturated with the same lipid mixture, which was discarded prior to the extrusion process. The size distribution and polydispersity index of the obtained SUVs were analyzed through Dynamic Light Scattering by using a Malvern Zetasizer Nano-Z (Malvern Instruments). For all the samples used, the average hydrodynamic diameter and polydispersity index were in the ranges 92–107 and 0.04–0.09 nm respectively.

### 4.3 Circular Dichroism Spectroscopy

CD spectra were recorded using a JASCO J-715 CD spectrometer equipped with a Peltier (PT-348WI) type cuvette holder at 20 °C, using a 1 mm path length quartz cuvette (Hellma Analytics 110-QS). The CD spectra were recorded using a bandwidth of 5 nm, a data pitch of 1 nm, a continuous scanning mode with speed of 20 nm/min, and a response time of 2 s. For each sample, CD spectra were recorded between 250 and 190 nm three times and averaged. The CD signal originating from the buffer (10 mM MES at pH 5.5) was then subtracted from the CD signal of the protein. The protein concentration in all samples was kept constant at 5 μM while the lipid concentration was varied accordingly for reaching the desired L/P. The CD signal was converted to mean residue ellipticity (MRE) and analyzed as a function of the L/P.

### 4.4 Aggregation Kinetics

Aggregation of αSyn was monitored with ThT fluorescence. 20 μM αSyn (in 10 mM MES, pH = 5.5) were mixed with 3 μM ThT, and aliquoted into a 96-well half area nontreated polystyrene plate (3880 Corning). The plate was sealed to avoid evaporation and incubated at 37 °C without shaking in a FLUOstar Omega plate reader (BMG Labtech, Offenburg, Germany). The aggregation was monitored by measuring excitation and emission wavelengths of 448 and 480 nm respectively, and with the cycle-time adjusted to the minimum-cycle-time to ensure constant mild agitation.^100^ Aggregation was followed until the fluorescence reached a plateau.

### 4.5 PICUP

PICUP was performed as described.^56^ The sample consisted of 20 μM αSyn in 10 mM MES, pH = 5.5, either by itself, or with added SUVs to a final ratio indicated for each experiment. 18 μL of that sample was then mixed with 1 μL of Ru­(bpy) (1 mM) and 1 μL of APS (20 mM). The mixture was exposed to 450 nm light for the desired time using a custom-made reaction chamber and stopped by addition of a 5× concentrated gel-loading dye.^56^ Samples were analyzed by sodium dodecyl sulfate polyacrylamide gel electrophoresis (SDS-PAGE) using Novex 10–20% Tricine precasted gels. PageRuler prestained protein ladder was used as a reference. Gels were stained overnight with InstantBlue and scanned with an Epson Expression 10000XL scanner.

## Supplementary Material



## Notes

Full gels of Figures 2A and 5B; PICUP of single Tyr → Phe αSyn mutants in the presence of SUVs; effect of lipid binding on PICUP of mutant; full gels of pictures shown in Figures 3 and 6; effect of ionic strength on PICUP of WT αSyn in solution; and estimating the dimensions of membrane-bound αSyn (PDF)

## Abbreviations

αSyn: α-synuclein
PICUP: photoinduced cross-linking of unmodified proteins
Nt: N-terminus
Ct: C-terminus
L/P: lipid-to-protein ratio
PvO: PICUP-visible oligomers
