# Supplementary material for: Transient Interactions of α‑Synuclein N- and C‑Termini
Source: ACS Chem Neurosci. 2026 Mar 30;17(8):1579–92. doi: 10.1021/acschemneuro.6c00108 (PMC13088187; doi:10.1021/acschemneuro.6c00108)
Supplement: Supplementary file 1 [file cn6c00108_si_001.pdf]

Supporting Information

for

Transient Interactions of α-Synuclein N- and C-Termini

Lei Ortigosa-Pascual*, Noemi Ferrante Carrante, Katja Bernfur, Katarzyna Makasewicz, Emma Sparr and Sara Linse*

Email: lei.ortigosa-pascual@kcl.ac.uk ; sara.linse@chem.lu.se

S1. Full gels of Figures 2A and 5B

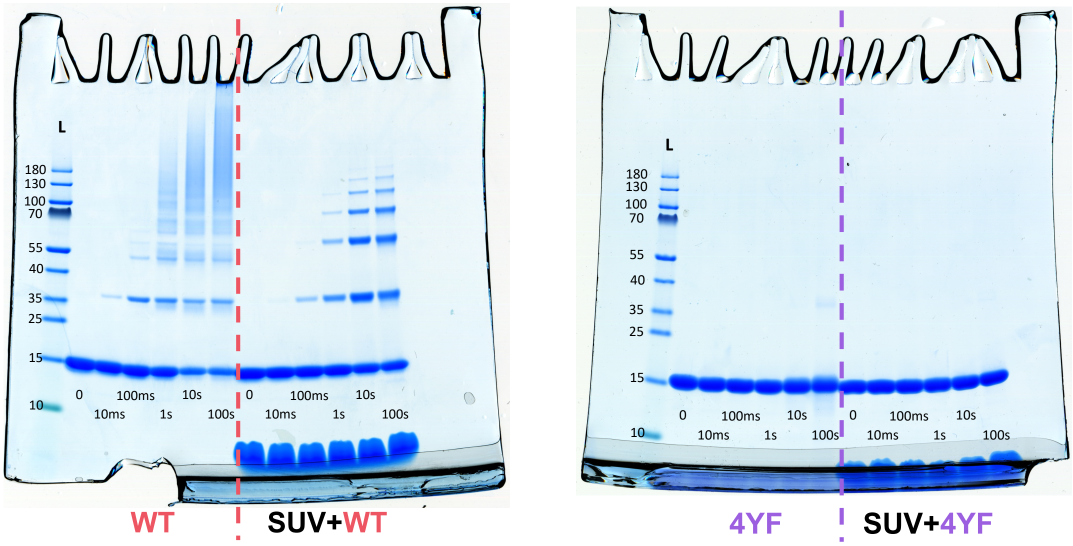

Figure S1. Full gels of pictures shown in Figure 2A and 5B. Cross-linking of WT and 4YF variant of αSyn, either without (left) or with (right) SUVs added, at a L/P ratio of 250. Lighting time is indicated in numbers below each lane.

S2. PICUP of single Tyr→Phe αSyn mutants in presence of SUVs

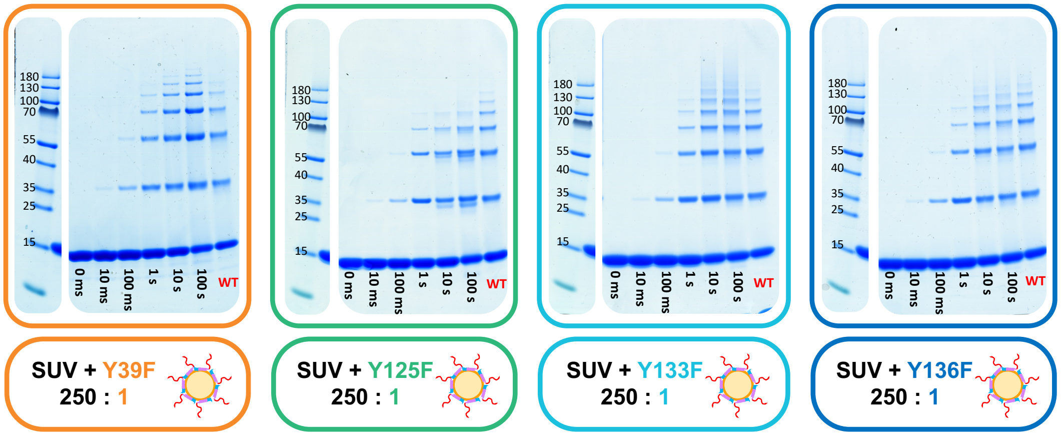

Figure S2. PICUP of single Tyr→Phe αSyn mutants. PICUP was performed for αSyn mutants Y39F, Y125F, Y133F and Y136F in the presence of SUVs at a L/P ratio of 250. The reaction was done for 0 ms, 10 ms, 100 ms, 1 s, 10 s and 100 s. Finally, a WT αSyn at L/P = 250 was cross-linked for 10 s and loaded in all gels as a reference (labelled WT, in red). Full gels can be seen in Figure S3.

S3. Effect of lipid binding on PICUP of mutant Y125F

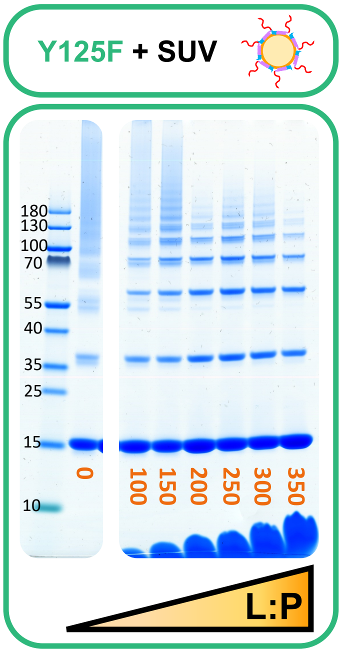

Figure S3. Effect of lipid binding on PICUP of mutant Y125F of αSyn. PICUP was performed to Y125F for 10 s in the presence of SUVs at different L/P (0 to 350), indicated at the bottom of each lane. Comparing these results to those observed for the WT αSyn (Figure 4A), Y125F requires a higher L/P ratio for the bands characteristic of PICUP in solution to disappear.

S4. Full gels of pictures shown in Figure 3 and 6

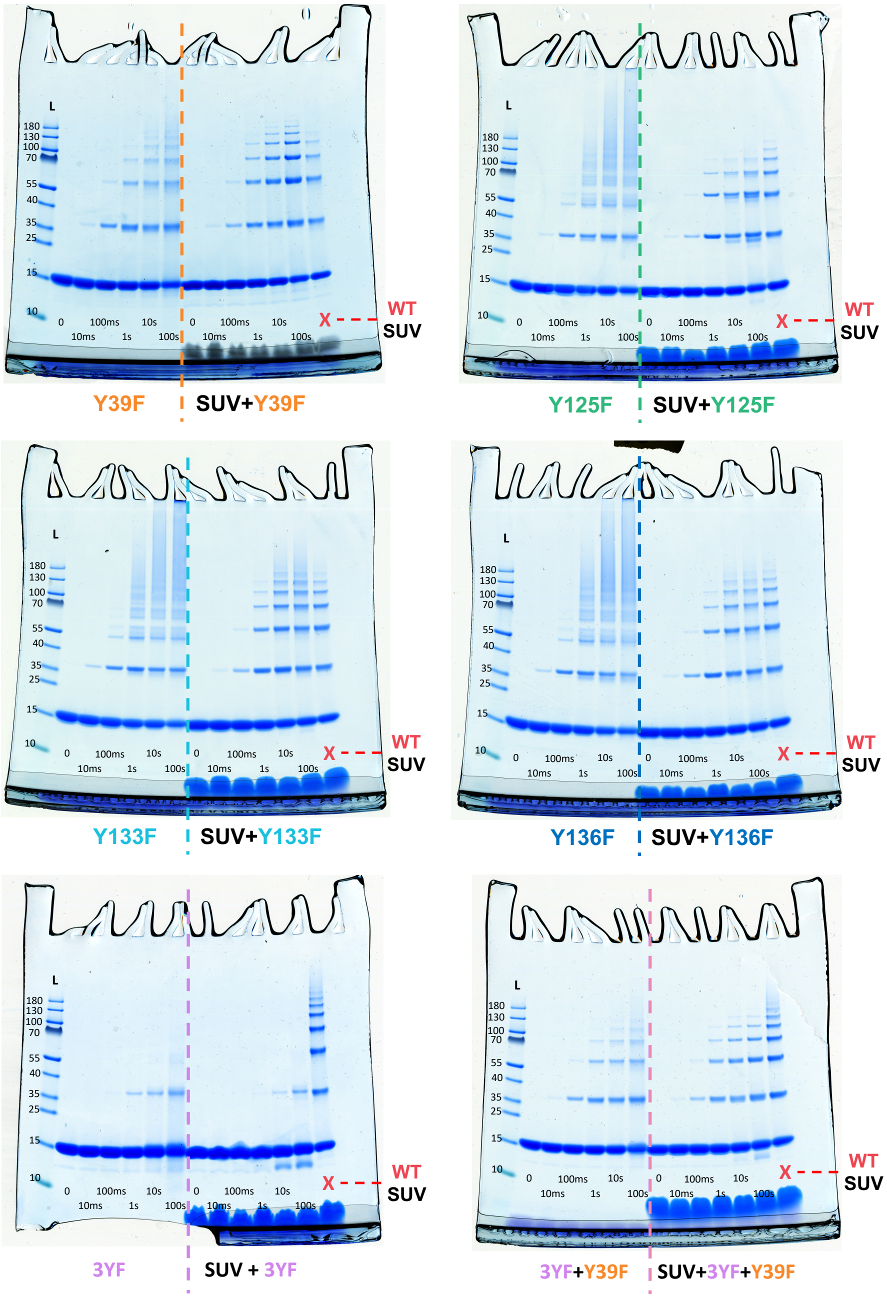

Figure S4. Full gels of pictures shown in Figure 3 and 6. Cross-linking of αSyn mutants Y39F, Y125F, Y133F, Y136F 3YF and a 50:50 ratio mixture of 3YF and Y39F, either without (left) or with (right) SUVs added, at a L/P ratio of 250. Lighting time is indicated in numbers below each lane. All gels contain a reference of WT αSyn sample in presence of SUV (L/P = 250), cross-linked for 10 s.

S5. Effect of ionic strength on PICUP of WT αSyn in solution

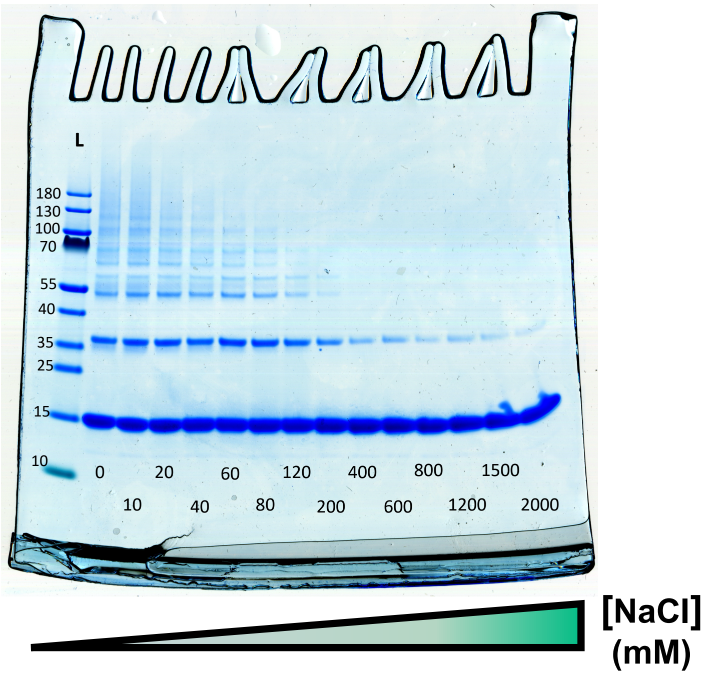

Figure S5. Effect of ionic strength on PICUP of WT αSyn in solution. PICUP was performed to αSyn for 1 s in the presence of different concentrations of NaCl (0 to 2000 mM).

S6. Estimating the dimensions of membrane-bound αSyn

To assess whether the Tyr at Ct could reach Y39 when αSyn is bound to membranes, we have calculated the distances between key points of the system. We have done this making the following assumptions:

a) Vesicles are unilamellar
b) αSyn only binds the outer leaflet of the vesicles
c) Lipids divide evenly between the outer and inner leaflet of the bilayer regardless of αSyn binding state
d) Essentially all proteins are membrane bound (1, 2)
e) The αSyn molecules are evenly distributed in the membrane

Given the protein concentration at the membrane at saturation (L/P ratio = 150, Figure 2C), and that only half the lipids would be in the outer leaflet of the bilayer, our system has the equivalent of 75 lipid molecules per protein molecule in the outer leaflet. Based on literature (3, 4), the area per lipid headgroup in the DOPC/DOPS (7/3) bilayer is of 70 $Å^2$. Thus, the average available area per protein, if evenly distributed at the membrane surface, is 75*70 = 5250 $Å^2$. This implies the average distance between two proteins at the membrane, and thus between two C-termini, is $\sqrt{5250}$ $Å^2$ ≈ 73 Å.

For a fully extended polypeptide, a length of 7.2 Å per every two amino-acids is estimated (5), corresponding to about 145 Å for the 40 residue Ct of αSyn. This is the maximum length the Ct can possibly reach. In this state, Y125, Y133 and Y136 would be at a distance of ~ 90 Å, ~119 Å and ~130 Å from L100, the residue where the Ct tail is "anchored".

If we instead consider the 40 residue Ct as a random coil, we can calculate the radius of gyration (R_g) of a random coil of N residues using equation $R_g = R_0 N^v$, where R_0 = 1.93 Å and v = 0.60, based on experiments and simulations (6). This gives us a R_g ~ 18 Å for a 40-residue segment.

We can calculate the dimensions of the first 100 residues of αSyn using the canonical dimensions of an α-helix (1.47 Å of rise per residue, 2.3 Å radius without sidechains (7)). This makes the length of the 100 residue α-helix 147 Å long, and the distance between L100 and Y39 ~ 90 Å.

Considering these distances, we have made a cartoon representation of the top view of αSyn bound to the surface of an SUV (Figure S6). The diagram made using the estimated dimensions shows that, while crowded, there is space for the Ct to reach the Y39 of the same αSyn molecule, and that the Tyr residues in the Ct can indeed reach Y39. This suggests that the reason why we don't see intraY39-Ct cross-linking for the membrane-bound αSyn is not steric hindrance, and points instead towards electrostatic repulsion between neighbouring Ct being the more likely cause.

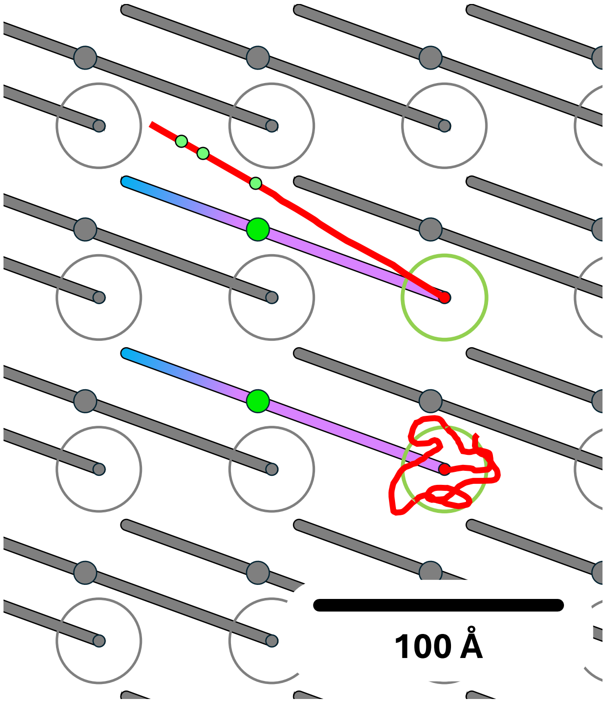

Figure S6. Cartoon representation of the top view of a SUV membrane with αSyn bound to its surface. The first 100 residues of αSyn, in α-helical form, are represented by a blue and purple bar, with Y39 as the green sphere on top of it. The Ct is colored red, with the top and bottom versions of it representing its fully extended and random coil conformations, respectively. The green circle with its center at the junction between the α-helix and the Ct shows the estimated radius of gyration of the Ct. The fully extended Ct on the top αSyn includes three green spheres at the equivalent position for Y125, Y133 and Y136. The features of the αSyn molecules neighboring the central two have been colored gray for ease of visualization.

References

(1) Makasewicz, K.; Wennmalm, S.; Stenqvist, B.; Fornasier, M.; Andersson, A.; Jonsson, P.; Linse, S.; Sparr, E. Cooperativity of α-Synuclein Binding to Lipid Membranes. *ACS Chemical Neuroscience* **2021**, *12* (12), 2099–2109. DOI: 10.1021/acschemneuro.1c00006

(2) Carrante, N. F.; Dubackic, M.; Makasewicz, K.; Wennmalm, S.; Hermodsson, T.; Bernfur, K.; Linse, S.; Sparr, E. α-Synuclein cooperative binding to lipid membranes is a robust property over a wide range of conditions. *Cell Reports Physical Science* **2025**, *6* (12), 103024. DOI: 10.1016/j.xcrp.2025.103024

(3) Petrache, H. I.; Tristram-Nagle, S.; Gawrisch, K.; Harries, D.; Parsegian, V. A.; Nagle, J. F. Structure and Fluctuations of Charged Phosphatidylserine Bilayers in the Absence of Salt. *Biophysical journal* **2004**, 86(3), 1574–1586. DOI: 10.1016/S0006-3495(04)74225-3

(4) Nagle, J. F.; Tristram-Nagle, S. Structure of lipid bilayers. *Biochimica et Biophysica Acta (BBA)-Reviews on Biomembranes* **2000**, 1469(3), 159-195. DOI: 10.1016/s0304-4157(00)00016-2

(5) Corey, R. B.; Pauling, L. C. Fundamental dimensions of polypeptide chains. *Proceedings of the Royal Society of London. Series B-Biological Sciences* **1953**, 141(902), 10–20. DOI: 10.1098/rspb.1953.0011

(6) Kohn, J. E.; Millett, I. S.; Jacob, J.; Zagrovic, B.; Dillon, T. M.; Cingel, N.; Dothager, R. S.; Seifert, S.; Thiyagarajan, P.; Sosnick, T. R.; Hasan, M. Z. Random-coil behavior and the dimensions of chemically unfolded proteins. *Proceedings of the National Academy of Sciences* **2004**, 101(34), 12491-12496. DOI: 10.1073/pnas.0403643101

(7) Pauling, L.; Corey, R. B.; Branson, H. R. The structure of proteins: two hydrogen-bonded helical configurations of the polypeptide chain. *Proceedings of the National Academy of Sciences* **1951**, 37(4), 205–211. DOI: 10.1073/pnas.37.4.205
